# Assessing Smoothness of Arm Movements With Jerk: A Comparison of Laterality, Contraction Mode and Plane of Elevation. A Pilot Study

**DOI:** 10.3389/fbioe.2021.782740

**Published:** 2022-01-21

**Authors:** Alexandra Roren, Antoine Mazarguil, Diego Vaquero-Ramos, Jean-Baptiste Deloose, Pierre-Paul Vidal, Christelle Nguyen, François Rannou, Danping Wang, Laurent Oudre, Marie-Martine Lefèvre-Colau

**Affiliations:** ^1^ AP-HP, Groupe Hospitalier AP-HP. Centre-Université de Paris, Hôpital Cochin, Service de Rééducation et de Réadaptation de l’Appareil Locomoteur et des Pathologies du Rachis, Paris, France; ^2^ Faculté de Santé, UFR Médecine Paris Descartes, Université de Paris, Paris, France; ^3^ INSERM UMR-S 1153, Centre de Recherche Épidémiologie et Statistique Paris Sorbonne Cité, ECaMO Team, Paris, France; ^4^ Institut Fédératif de Recherche sur le Handicap, Paris, France; ^5^ Centre Giovanni Alfonso Borelli, ENS Paris-Saclay, Université Paris-Saclay, CNRS, Gif-Sur-Yvette, France; ^6^ Machine Learning and I-health International Cooperation Base of Zhejiang Province, Hangzhou Dianzi University, Hangzhou, China; ^7^ Department of Neurosciences, Universitá Cattolica del SacroCuore, Milan, Italy; ^8^ INSERM UMR-S 1124, Toxicité Environnementale, Cibles Thérapeutiques, Signalisation Cellulaire et Biomarqueurs (T3S), Faculté des Sciences Fondamentales et Biomédicales, Université de Paris, Paris, France; ^9^ Plateforme Sensorimotricité, BioMedTech Facilities INSERM US36-CNRS UMS2009-Université de Paris, Paris, France

**Keywords:** jerk quantification, smoothness analysis, IMU’s data processing, arm elevation, functional movement, laterality, elevation plane, contraction mode

## Abstract

Measuring the quality of movement is a need and a challenge for clinicians. Jerk, defined as the quantity of acceleration variation, is a kinematic parameter used to assess the smoothness of movement. We aimed to assess and compare jerk metrics in asymptomatic participants for 3 important movement characteristics that are considered by clinicians during shoulder examination: dominant and non-dominant side, concentric and eccentric contraction mode, and arm elevation plane. In this pilot study, we measured jerk metrics by using Xsens^®^ inertial measurement units strapped to the wrists for 11 different active arm movements (ascending and lowering phases): 3 bilateral maximal arm elevations in sagittal, scapular and frontal plane; 2 unilateral functional movements (hair combing and low back washing); and 2 unilateral maximal arm elevations in sagittal and scapular plane, performed with both arms alternately, right arm first. Each arm movement was repeated 3 times successively and the whole procedure was performed 3 times on different days. The recorded time series was segmented with semi-supervised algorithms. Comparisons involved the Wilcoxon signed rank test (*p* < 0.05) with Bonferroni correction. We included 30 right-handed asymptomatic individuals [17 men, mean (SD) age 31.9 (11.4) years]. Right jerk was significantly less than left jerk for bilateral arm elevations in all planes (all *p* < 0.05) and for functional movement (*p* < 0.05). Jerk was significantly reduced during the concentric (ascending) phase than eccentric (lowering) phase for bilateral and unilateral right and left arm elevations in all planes (all *p* < 0.05). Jerk during bilateral arm elevation was significantly reduced in the sagittal and scapular planes versus the frontal plane (both *p* < 0.01) and in the sagittal versus scapular plane (*p* < 0.05). Jerk during unilateral left arm elevation was significantly reduced in the sagittal versus scapular plane (*p* < 0.05). Jerk metrics did not differ between sagittal and scapular unilateral right arm elevation. Using inertial measurement units, jerk metrics can well describe differences between the dominant and non-dominant arm, concentric and eccentric modes and planes in arm elevation. Jerk metrics were reduced during arm movements performed with the dominant right arm during the concentric phase and in the sagittal plane. Using IMUs, jerk metrics are a promising method to assess the quality of basic shoulder movement.

## 1 Introduction

The upper limb is designed for basic (daily life activities) and specific human needs related to relational life, sports and art (Doorenbosch et al., 2003; Dufour, 2005). Because of the wide diversity and large range of motion (RoM) of arm movements, their assessment is challenging, notably in routine clinical examination (Rau et al., 2000; Caimmi et al., 2015).

Clinicians need accurate, reliable and easily available information on arm position and RoM to assess movement related impairments, choose an intervention, evaluate its efficacy and monitor the follow-up of patients (Skirven et al., 2011; Braito et al., 2018). Nevertheless, this measurement is challenging because it involves both quantitative (mobility, muscular strength and endurance) and qualitative (precision and smoothness) abilities and stretches the limits of classical clinical tests (Skirven et al., 2011; Braito et al., 2018).

Recently, with the evolution of technology and the miniaturization of devices, high-quality, affordable and wearable inertial measurement units (IMUs) have become available to assess movements (Patel et al., 2012; Wang et al., 2017; Vienne-Jumeau et al., 2020). IMUs use a combination of accelerometer, gyrometer, and magnetometer sensors combined with sensor-fusion algorithms to measure the 3D orientation of the system of reference and then estimate joint kinematics with accuracy (Zhu and Zhou, 2004; De Baets et al., 2017). IMUs are increasingly being used for assessing arm movement and for arm rehabilitation (Wang et al., 2017).

The smoothness of a movement is a characteristic linked to its regularity (i.e., to the variation of its velocity) that appears difficult to quantify in a clinical context, in comparison with RoM or execution time for instance. The development of metrics to quantify the smoothness of the movement dates back to 1985 (Flash and Hogan, 1985), and has been an active research field since (Gulde and Hermsdörfer, 2018). Three main categories of parameters have been proposed: velocity parameters, acceleration parameters, and arc-length parameters. Velocity parameters, such as number of velocity peaks or the normalized average speed, focus on the evolution of the speed of the body parts (Rohrer et al., 2002). Arc length parameters, such as the speed arc length or the spectral arc length, measure the lengths of other parameters trajectories when observed in a well chosen space (either speed or Fourier) (Balasubramanian et al., 2011). Acceleration parameters, such as the regular jerk, or it’s normalized versions such as the normalized jerk or the log-dimensionless jerk, take into consideration the quantity of acceleration variation of the body parts in order to estimate a notion of regularity (Balasubramanian et al., 2011).

The jerk is defined as the time rate of change in acceleration or the quantity of acceleration variation. A minimal jerk trajectory corresponds to a trajectory for which the kinematic parameters (position, speed, and acceleration) vary in the most continuous, regular manner over time. Minimum jerk reflects maximal smoothness. Most studies used jerk to assess tasks involving planar and limited shoulder RoM and fine coordination of the upper-limb joints in asymptomatic individuals and patients with neurologic conditions (Smith et al., 2000; Hogan and Sternad, 2009). The jerk can be well estimated by using the accelerometers included in IMUs (Pan et al., 2020). Using IMUs, jerk has been used to quantify arm movement smoothness in asymptomatic individuals and patients with neurologic conditions. In asymptomatic individuals, studies assessed lifting performance (Tammana et al., 2018) and specific sports movement (baseball overhead pitching) (Lapinski et al., 2019). With patients, measuring movement smoothness using jerk helped assess arm function in multiple sclerosis (Carpinella et al., 2014) and in children with hemiparesis (Newman et al., 2017) as well as spontaneous upper-limb motion in acute stroke patients (Datta et al., 2021) and the finger-to-nose test in subacute stroke patients (Chen et al., 2021) and characterize bilateral manual arm use among stroke survivors (de Lucena et al., 2017) and children with unilateral cerebral palsy (Pan et al., 2020). The studies using jerk metrics focused on the association between upper extremity motor function and jerk. The jerk has never been used to identify the characteristics of basic shoulder movements. In the current study, the constrained duration of the movements and the similarity of the compared trajectories allow to disregard the issue of metric normalization, and therefore to use the regular jerk.

Hand dominance, contraction mode and plane of elevation are 3 important parameters that characterize arm movement. These characteristics are considered by clinicians during their examination of the shoulder complex that includes visual comparative assessment of arm analytic and functional movements. The dominant hand was found more performant for tasks involving speed and dexterity (Roy et al., 2003; Scharoun and Bryden, 2014). Eccentric (lengthening) contractions (occurring during the lowering phase of arm elevation) involve reduced muscle activation as compared with concentric contractions [during the ascending phase of arm elevation (Franchi et al., 2017; Hody et al., 2019)]. The scapular plane of arm elevation (about 40° in front of the frontal plane) is supposed to be the most biomechanically advantageous, the axis of the humerus being aligned with the axis of the spine of the scapula reducing mechanical constraints and providing glenohumeral stability by the humeral head centering in the glenoid cavity (Ozaki and Kawamura, 1984; Lefèvre-Colau et al., 2018).

Considering the available knowledge on hand dominance, concentric and eccentric contractions, planes of movement, and jerk, we formulated the following hypotheses: jerk is reduced during arm elevation with the dominant side, during the ascending phase of arm elevation (concentric muscular mode of contraction) and during arm elevation in the scapular plane. The main objective of the current study was to assess motion smoothness using jerk, according to major movement characteristics, during bilateral and unilateral analytic arm elevation and during 2 functional movements in asymptomatic participants.

## 2 Materials and Methods

We conducted a single-center pilot observational study on asymptomatic participants. Inclusion criteria were: age ≥18 years, no pathology or history of pathology or surgery affecting the spine or the upper limb, and agreement to participate in the study. Exclusion criteria were uncompensable visual deficit.

The study protocol was conducted in compliance with the Good Clinical Practices protocol and Declaration of Helsinki principles. All participants provided informed written consent. STROBE (Von Elm et al., 2014) and GRRAS (Kottner et al., 2011) guidelines were used for reporting.

### 2.1 Material

The measurements were performed in a French tertiary care center from May to July 2019. We used 4 Mtw Awinda XSens^®^ (weight 16 g, dimensions 47 mm × 30 mm × 13 mm, sensitivity ± 2000 deg/s and ± 160 m/s^2^) (XSens^®^ Technologies, Enschede, the Netherlands). All signals were acquired at 100 Hz with the Awinda Recording and Docking Station.

### 2.2 Experimental Setup

**FIGURE 1:**
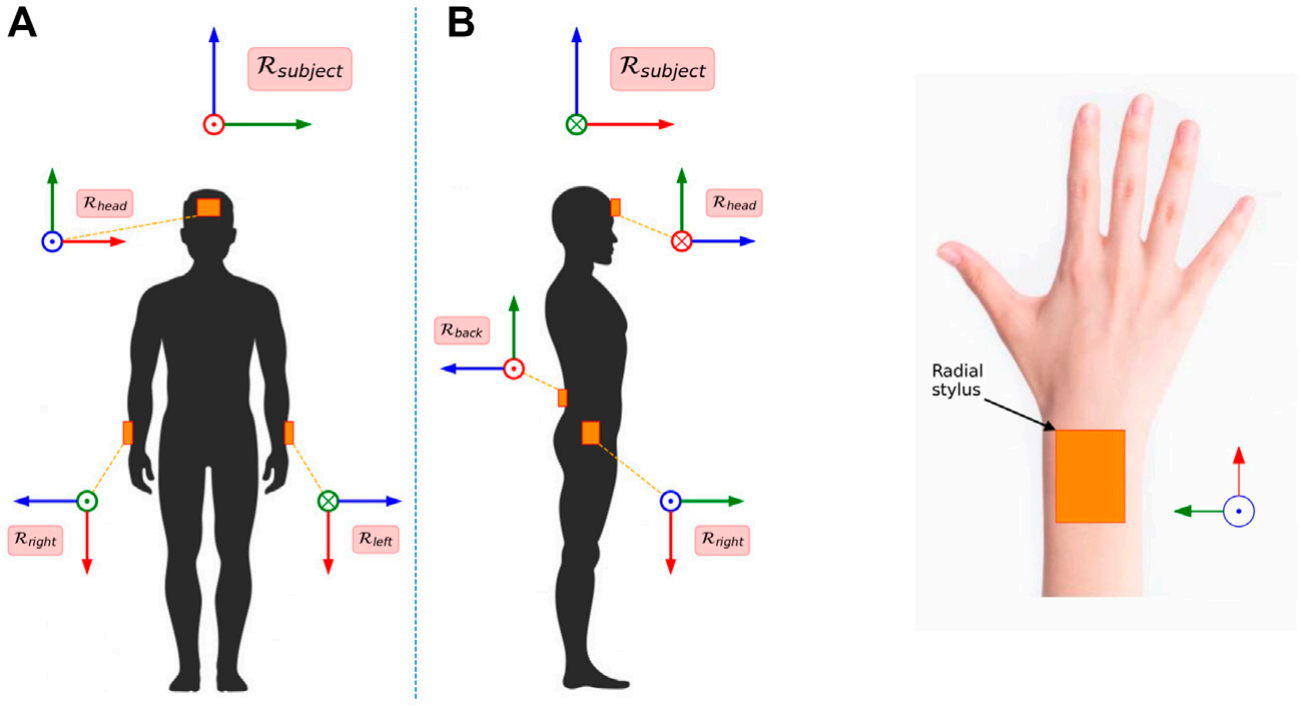
Sensor position on the upper body **(A)** and sensor position on the right wrist **(B)**. Colors red, green and blue correspond to axes X, Y, and Z of the sensors, respectively. The data from the forehead sensor and the back sensor were not included in the present analysis.

Participants were in a 10 m^2^ room. Four sensors were strapped with Velcro on the right and left wrist (the lower limit of the wrist strap was at the radial stylus level), on the forehead (the lower limit of the strap was 2 cm above the eyebrows and on the lower back (lumbosacral level) (Figure 1A). During each recording session, the participant performed 11 arm movements (analytic and functional) that were chosen to reflect shoulder clinical examination (Table 1).

**TABLE 1:** List of the movements performed during a recording session, presented in execution order.

	Arm movement	Functional or analytic	Position	Bilateral or unilateral right or left
1	Sagittal plane elevation	Analytic	Seated	Bilateral
2	Scapular plane elevation	Analytic	Seated	Bilateral
3	Frontal plane elevation	Analytic	Seated	Bilateral
4	Hair combing	Functional	Seated	Unilateral right
5	Hair combing	Functional	Seated	Unilateral left
6	Low back washing	Functional	Seated	Unilateral right
7	Low back washing	Functional	Seated	Unilateral left
8	Sagittal plane elevation	Analytic	Standing	Unilateral right
9	Sagittal plane elevation	Analytic	Standing	Unilateral left
10	Scapular plane elevation	Analytic	Standing	Unilateral right
11	Scapular plane elevation	Analytic	Standing	Unilateral left

Participants were asked to face a large height-adjustable target on the wall in front of them at eye level during each arm movement to standardize the position of the head among participants. They were instructed to complete each analytic arm movement for about 10 s. To help the participants, the observer counted out loud.

For the first part of the experiment, participants were seated on a fixed stool, arms alongside the body, straight back, feet flat on the floor (Supplementary Appendix Figures SA1A,B). First, they performed bilateral maximal arm elevation (ascending and lowering phase), elbows in extension, in sagittal, scapular and frontal planes, successively, (Supplementary Appendix Figure SA1C). Then, they performed functional movement [simulation of 2 activities of daily living (ADL)]: low back washing (Supplementary Appendix Figure SA1D) and hair combing (Supplementary Appendix Figure SA1E) successively. These 2 ADL have been frequently investigated in studies assessing shoulder motion (Sheikhzadeh et al., 2008; Rundquist et al., 2009). They are frequent movements contributing to autonomy and they involve combined movements at the glenohumeral joint: scapular plane arm elevation and lateral rotation for hair combing, extension and medial rotation for low back washing. For hair combing, the instruction was to raise the hand in front of the face, then pass it above the head and finally to go down to the nape of the neck. For low back washing, the instruction was to touch the lower back (above the sensor) with the back of the hand. The participants were instructed to avoid touching the lower back sensor. Functional movements were performed with both arms alternately, right arm first.

For the second part of the experiment, participants were standing and performed unilateral maximal arm elevation (ascending and lowering phase) in sagittal and scapular plane, successively (Supplementary Appendix Figure SA1F). Participants performed each arm elevation with both arms alternately, right arm first.

For analytic arm movement, no instruction was given about the position of the hand (i.e., the rotation of the humerus).

Before recording, participants practiced the motion at least once. During analytic arm movement, the observer visually checked the plane of the movement by using the projection of a light beam (attached by Velcro on the wrist cuff) on marks on the wall. The observer checked the good quality of each movement performed. If a movement was considered incorrect (incomplete movement, wrong speed, or wrong arm elevation plane), it was repeated. The arm elevations and functional movements assessed in the current study were part of a longer protocol containing 6 other arm movements. The whole protocol lasted about 30 min; the first part, presented in the current study, lasted about 20 min.

**FIGURE 2:**
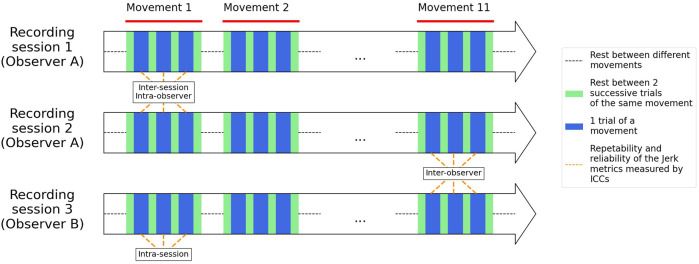
Example of a timeline of the full protocol for 1 participant (the participants has 3–7 days rest between each recording session). The observers were randomized.

To assess the intra-(recording) session repeatability, each arm movement was performed 3 times successively (3 trials) with a rest time of 3 s between each trial. Participants had a rest time of 10 s between each different movement (Figure 2). To assess inter-session reliability, each participant performed the whole experimental procedure (i.e., recording session), 2 with observer A (JBD) and 1 with observer B (DVR) (observers A and B are physiotherapists experienced in shoulder assessment and the use of IMUs). The trial-observer was randomized. The participant had 3–7 days’ rest between each recording session.

The position of the participant (seated or standing) was not considered in the present study.

### 2.3 Data Pipeline

The Xsens MVN studio software (MVN Studio, Xsens, the Netherlands) was used for data capture. The measurements were performed at 100 Hz and synchronized over the 4 sensors. The following time series were measured by the sensors:

• Sensor free linear accelerations in the sensor frames ( $R_{\text{right}}$ , $R_{\text{left}}$ , $R_{\text{head}}$ and $R_{\text{back}}$ ).
• Sensor angular velocities.
• Sensor orientations in an initial (random) common frame.

Free accelerations are defined as the accelerations measured by the sensor minus the effect of gravity. In the current study, all presented accelerations were free accelerations. The accelerations and angular velocities were measured raw by the sensors, and the orientation was estimated with a Kalman filter (Xsens Kalman Filter, XKF). A subject frame $R_{\text{subject}}$ , such that the *x* axis is orthogonal to the frontal plane and the *z* axis is vertical, was obtained from the subject’s resting position (Figure 1): the vertical (z) axis is already provided by the Xsens software, and the *y* axis is obtained from the orientations of the forehead and lower back sensors, from which the vertical component has been removed. After frame transformation, the orientations take the form of a rotation matrix time series that characterizes the orientation of the sensor frames $R_{\text{right}}$ , $R_{\text{left}}$ , $R_{\text{head}}$ and $R_{\text{back}}$ with regard to the subject frame $R_{\text{subject}}$ . The orientations are displayed in Supplementary Appendix Figure SA2A.

From these inputs, the following 4 quantities were computed:

• Left/right wrist linear acceleration **a** ^left^(*t*)/**a** ^right^(*t*): the 3D free linear accelerations in the sensor’s frames. Assuming that the sensor was rigidly attached, this quantity can be assimilated with the acceleration of the body part it was attached to. For a sensor “s” (“left” or “right”), the acceleration consists of the linear accelerations projected on the 3 axes of the sensor frame $a_{\text{x}}^{\text{s}}\left(t\right)$ , $a_{\text{y}}^{\text{s}}\left(t\right)$ and $a_{\text{z}}^{\text{s}}\left(t\right)$ :

$$a^{\text{s}}\left(t\right)=\left[\begin{array}{c} a_{\text{x}}^{\text{s}}\left(t\right)\\ a_{\text{y}}^{\text{s}}\left(t\right)\\ a_{\text{z}}^{\text{s}}\left(t\right) \end{array}\right]\in R^{3}$$ (1)

• Left/right wrist acceleration norm ‖**a** ^left^(*t*)‖/‖**a** ^right^(*t*)‖: given the linear acceleration signals of a sensor “s,” $a_{\text{x}}^{\text{s}}\left(t\right)$ , $a_{\text{y}}^{\text{s}}\left(t\right)$ and $a_{\text{z}}^{\text{s}}\left(t\right)$ , the norm of the acceleration vector represents the total quantity of acceleration:

$$\|a^{\text{s}}\left(t\right)\|=\sqrt{a_{\text{x}}^{\text{s}}{\left(t\right)}^{2}+a_{\text{y}}^{\text{s}}{\left(t\right)}^{2}+a_{\text{z}}^{\text{s}}{\left(t\right)}^{2}}$$ (2)

• Left/right wrist elevation angle $\theta_{\text{E}}^{\text{left}}\left(t\right)$ / $\theta_{\text{E}}^{\text{right}}\left(t\right)$ : the angle between the *z*-axis of $R_{\text{subject}}$ , oriented downward, and the *x*-axis of the right/left sensor. This quantity is positive, ranging from 0° when the arm is pointing downward, to 180° when the arm is pointing upward.
• Left/right wrist angle in the subject horizontal plane $\theta_{\text{H}}^{\text{left}}\left(t\right)$ / $\theta_{\text{H}}^{\text{right}}\left(t\right)$ : this quantity was computed by first projecting the sensor *x*-axis onto the subject horizontal plane, then computing the signed angle between the subject *x*-axis and the projection. This angle allowed us to differentiate the plane in which the elevation was performed. Of note, this quantity estimation is irrelevant if the angular elevation $\theta_{\text{E}}^{\text{s}}\left(t\right)$ is too low or too high because the notion of angle in the plane becomes ill-defined when the axis is orthogonal to that plane. In this study, we considered this quantity only when the angular elevation ranged from 30° to 150°.

**FIGURE 3:**
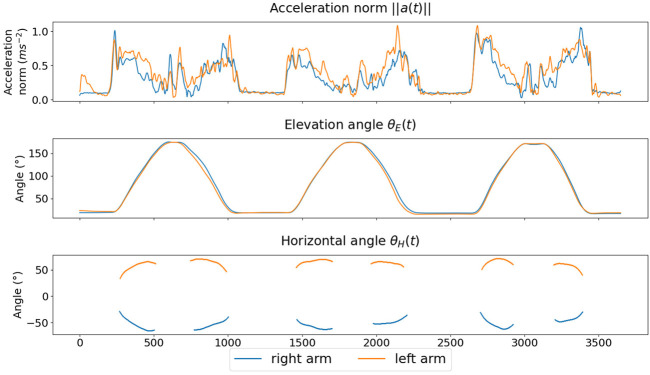
Example of signals for a participant performing scapular plane arm elevation.

The different angles introduced in this paragraph are displayed in Supplementary Appendix Figure SA2B, and all the presented quantities are displayed during a movement as time series in Figure 3. In accordance with previous research, all the resulting time series were pre-processed with a fourth-order Butterworth filter with cutoff frequency of 10 Hz (Zhou et al., 2008).

### 2.4 Time Series Segmentation

Because each recorded time series consisted of three successive trials of the same movements, the signals presented in the previous subsection needed to be segmented to isolate the distinct trials. This time series segmentation task was performed with a 4 step pipeline:

• The manual segmentation of all the time series for 20% of the subjects.
• A decision random forest training, which estimated for each timestep the probability of being a motion timestep or a resting timestep. The decision tree was trained on the manually segmented signals. The entry signals of the decision tree were the right and left wrist acceleration norms (‖**a** ^left^(*t*)‖ and ‖**a** ^right^(*t*)‖) and the wrist elevation angles $\theta_{E}^{\text{left}}\left(t\right)$ and $\theta_{E}^{\text{right}}\left(t\right)$ .
• The decision random forest fit on the remaining 80% of the time series. The resulting signals were the time series of motion probability, denoted as *p*_motion_(*t*). For a timestep *t* during the movement, *p*_motion_(*t*) ∼ 1, whereas for a resting timestep *t*, *p*_motion_(*t*) ∼ 0.
• The segmentation of the signal *p*_motion_(*t*) into 7 different phases: 4 resting phases and 3 movement phases. The segmentation was performed with the package Ruptures (Truong et al., 2020). Additional constraints were applied to the algorithm: the segmentation sought needed to consist of resting phases [i.e., low *p*_motion_(*t*) segments] and movement phases [i.e., high *p*_motion_(*t*) segments] alternately. Each segment duration was imposed to be more than 1 s and less that 20 s.

**FIGURE 4:**
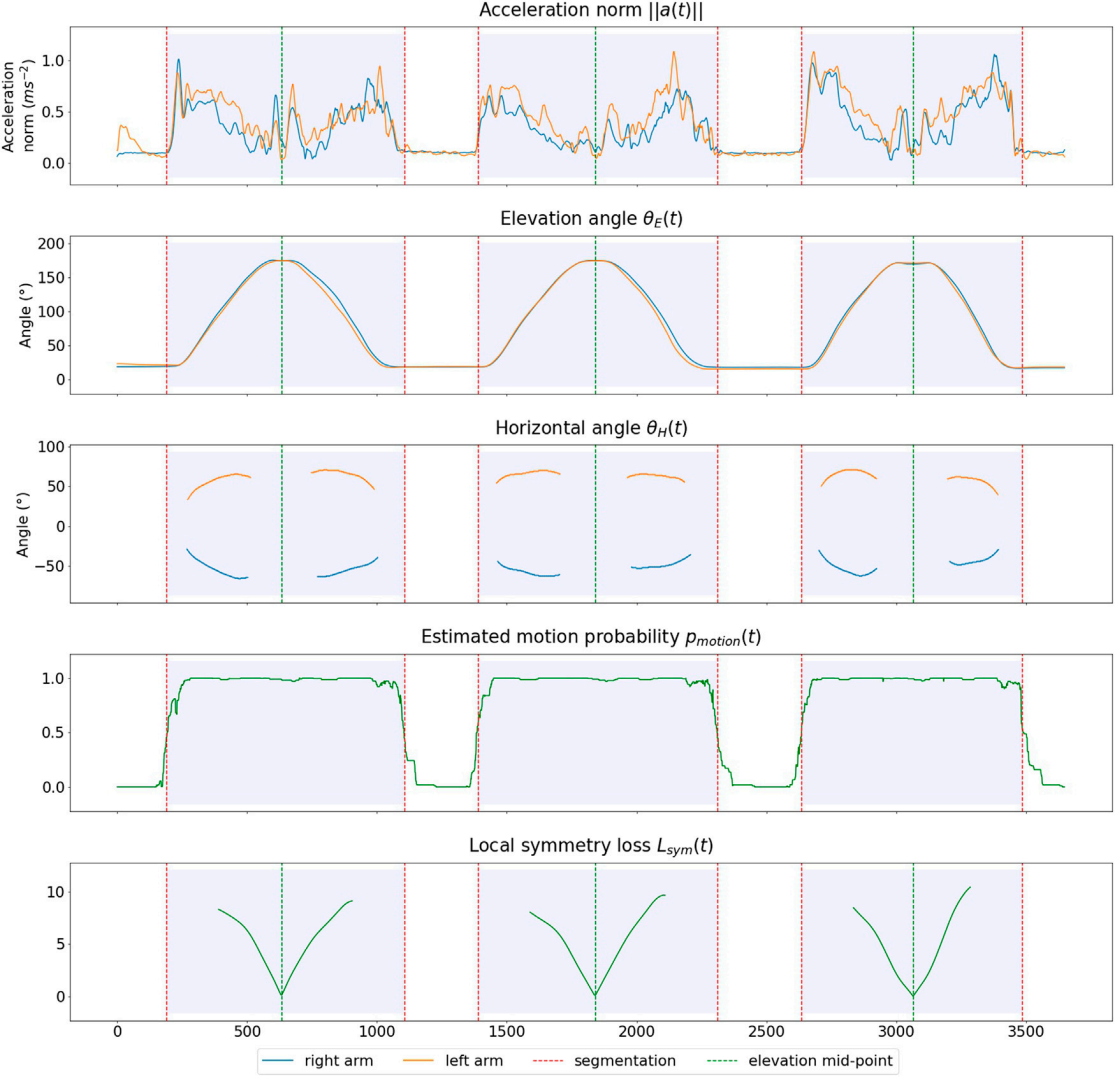
Example of computed segmentation and midpoints for a participant performing scapular plane arm elevation.

The decision random forest was trained with the sklearn.ensemble.RandomForestClassifier algorithm, along with *n* = 40 estimators. The resulting model achieved 94*%* accuracy on the training dataset. The Ruptures package gathers a wide panel of multivariate time series segmentation methods along with the possibility to implement personalized loss functions, therefore allowing custom conditions on the resulting segmentation. Figure 4 displays the signals for a scapular arm elevation, the estimated motion probability signal and the resulting segmentation.

The present study required comparing jerk metrics between the ascending phase and lowering phase of an arm elevation, which depends upon a mid-elevation timestep. Our approach for estimating the timestep of mid-elevation was based on a local symmetry criterion of the elevation angles *θ*_*E*_(*t*). For each candidate timestep, we computed a local symmetry loss *L*_*sym*_(*t*) (3), which quantified how symmetric the signal was on the interval [*t* − *w*, *t* + *w*] (with *w*, the window size, set to 100 timesteps). The timestep of mid-elevation was chosen to minimize the symmetry loss. Figure 4 displays the symmetry loss of candidate timesteps as well as the resulting mid-elevation timestep.

$$\begin{array}{cc} L_{\text{sym}}^{\text{s}}\left(t\right) & =\sqrt{\frac{1}{w}\overset{w}{\underset{i=1}{\sum }}{\left[\theta_{\text{E}}^{\text{s}}\left(t-i\right)-\theta_{\text{E}}^{\text{s}}\left(t+i\right)\right]}^{2}}\\ L_{\text{sym}}\left(t\right) & =\sqrt{{\left[L_{\text{sym}}^{\text{right}}\left(t\right)\right]}^{2}+{\left[L_{\text{sym}}^{\text{left}}\left(t\right)\right]}^{2}} \end{array}$$ (3)

### 2.5 Computation of Jerk Metrics and the Jerk Ratio

Given the trajectory of a point, $r\left(t\right)\in R^{3}$ , between the starting timestep *t*_init_ and the ending timestep *t*_end_, the jerk quantity is defined as follows (Flash and Hogan, 1985):

$$\text{Jerk Function}=\int_{t_{\text{init}}}^{t_{\text{end}}}\|\frac{\partial^{3}r}{\partial t^{3}}\left(t\right){\|}^{2}\text{d}t$$ (4)

The previous expression is introduced as a function of the position, and the sensors provided the local free acceleration (i.e., the second-order derivative of the position as a discrete time series).

#### 2.5.1 Jerk Metrics

For a specific sensor denoted as “s” (right, left, head, or back), the jerk quantity was computed from the free acceleration of the sensor according to the following formula:

$$J_{s}\left(t_{init},t_{end}\right)=\sqrt{\overset{t_{\text{end}}}{\underset{t=t_{\text{init}}}{\sum }}\|a^{s}\left(t+1\right)-a^{s}\left(t\right){\|}^{2}}$$ (5)

From the jerk definition for the trajectory of a point, we define the jerk of the total motion for any upper-limb movement as follows:

$$J_{\text{total}}\left(t_{\text{init}},t_{\text{end}}\right)=\sqrt{{\left[J_{\text{left}}\left(t_{\text{init}},t_{\text{end}}\right)\right]}^{2}+{\left[J_{\text{right}}\left(t_{\text{init}},t_{\text{end}}\right)\right]}^{2}}$$ (6)

The resulting metric quantifies the fusion of the jerks for the left arm and right arm as a whole.

#### 2.5.2 Jerk Ratio

The analysis performed in this study aimed at demonstrating that some compatible jerk metrics can be ordered (i.e., that comparable trajectories were performed with significantly different jerk quantities). However, without prior knowledge of the range values taken by the jerk metrics, our analysis required a clear and normalized quantification of such differences.

For this aim, we introduced the notion of jerk ratio, which allows the comparison of 2 jerk quantities. Given 2 jerk quantities, *J*_1_ and *J*_2_, the jerk ratio for motion 1 against motion 2 is defined as follows:

$$r_{\left(1\right)/\left(2\right)}=\frac{J_{\left(1\right)}}{J_{\left(1\right)}+J_{\left(2\right)}}$$ (7)

This ratio allowed for the normalized comparison of 2 jerk metrics. A ratio ≥0.5 indicates a higher *J*_(1)_ relative to *J*_(2)_, whereas a ratio ≤0.5 corresponds to a lower *J*_(1)_ relative to *J*_(2)_. The computation and analysis of these ratios allows us to quantify how strong the jerk difference is (i.e., a jerk ratio of 0.1 corresponds to a strong difference, and a ratio of 0.45 corresponds to a small difference) with a normalized quantity.

### 2.6 Statistical Analysis

Considering the novelty of the comparisons of jerk metrics between different conditions of active analytic and functional arm movements, we were not able to perform a power analysis. Based on previous studies of jerk metrics the sample size of 30 participants appears to be appropriate (Pan et al., 2020; Chen et al., 2021). This sample size is also suitable for a reliability study (Fermanian, 1984).

R was used for the statistical analysis, along with the packages “psych” and “irr.” The variables of interest for each subject were the jerk metrics for each arm elevation trial (right or left arm or both arms together, for ascending or lowering phase or both phases together) and the jerk metrics for each functional movement (right or left arm). Intra-session trial-to-trial repeatability and inter-session intra- and inter-observer reliability were assessed with the intraclass correlation coefficient (ICC) based on the two-way random effects ANOVA for a single measurement [ICC (3,1) for intrasession and ICC (2,1) for intersession] (Shrout and Fleiss, 1979). The repeatibility/reliability was determined with the following criteria: < 0.40 = poor; 0.40–0.59 = fair; 0.60–074 = good; > 0.74 = excellent.

In addition, the precision of the measurements was assessed using the standard error of measurement (SEM) and the 95% minimal detectable change (MDC) (Atkinson and Nevill, 1998; de Vet et al., 2006).

For unbiased comparison tests for the recorded population, only one data point per subject was included in the statistical analysis. The 3 recording sessions performed by each subject and the 3 trials of each movement lead to 9 values for each studied jerk metrics. We considered the median of these values.

We compared the following jerks using the jerk ratios:

• The right jerk against the left jerk during arm elevation for all arm elevations, with *r*_(Left)/(Right)_.
• The ascending jerk against the lowering jerk for bilateral and unilateral arm elevations, with *r*_(Ascending)/(Lowering)_.
• The jerk of arm elevations in the three different planes (Frontal, Sagittal, and Scapular). The comparisons involved the pairwise jerk ratio [*r*_(Front)/(Sag)_, *r*_(Front)/(Scap)_ and *r*_(Sag)/(Scap)_].

Functional movements were excluded from comparisons of jerk metrics between ascending and lowering phases of the movement because we could not determine with accuracy the mid point of the motion (i.e., the moment of the transition between ascending and descending phases). Functional movements were also excluded from comparisons of jerk metrics between the 3 planes of arm elevation because those movements involve several planes of elevation.

The position of the participant (seated or standing) were not taken into account in the present study. The jerk quantities of the Forehead sensor and the Lower back sensor were not computed and not included in the present analysis. The comparisons involved a Wilcoxon mean comparison test (*p* ≤ 0.05). All presented *p*-values were corrected with the Bonferroni correction method.

## 3 Results

### 3.1 Participants

A convenience sample of 30 right-handed asymptomatic volunteers was included between May and July 2019 [17 men (57%), mean (SD) age 31.9 (11.4) years, mean BMI 22.7 (3.1) kg.m^2^]. The participants belonged to the professional or student circle of the authors and were asked to join the study by personal communication. All subject provided written informed consent. The entire procedure (3 tests) was performed over a mean of 14.5 (6.4) days. Because of an experimental error, one of the measurements was performed at 40 Hz, so only the measurements for 29 of 30 participants were used for the study. The distribution of the participants’ jerk metrics is illustrated in Supplementary Appendix Figures SA3–A5.

### 3.2 Reliability

For intra-session trial-to-trial repeatability, the ICC ranged from 0.55 to 0.93 for analytic arm elevation and 0.89 to 0.95 for functional movements. For inter-session intra-observer reliability, the ICC ranged from 0.19 to 0.76 for analytic arm elevation and from 0.44 to 0.65 for functional movements. For inter-session inter-observer reliability, the ICC ranged from 0.19 to 0.78 for analytic arm elevation and from 0.64 to 0.89 for functional movements (Table 2). Overall, for inter-session reliability, the smallest ICC (0.19) was for the lowering (eccentric) phase of arm elevation and the highest ICC (0.89) was for hair combing. On the whole, the SEM ranged from 0.07 m s^−2^ (MDC = 0.19 m s^−2^) to 1.38 m s^−2^ (MDC = 3.83 m s^−2^) (Tables 2, 3). The jerk mean values ranged from 0.56 m s^−2^ to 5.61 m s^−2^.

**TABLE 2:** Intra-session repeatability, intersession intra and inter observer reliability and agreement of jerk metrics for all arm movements.

Movements		Total jerk for the right arm	Total jerk for the left arm	Jerk for ascending phase of right arm elevation	Jerk for lowering phase of right arm elevation	Jerk for ascending phase of left arm elevation	Jerk for lowering phase of left arm elevation	Total jerk for the both arm
ICC (SEM/MDC) for intra-session trial to trial repeatability								
Analytic arm movement	Bilateral sagittal arm elevation	0.83 (0.29/0.82)	0.88 (0.25/0.69)	0.87 (0.23/0.63)	0.89 (0.09/0.26)	0.61 (0.21/0.60)	0.88 (0.13/0.35)	0.87 (0.16/0.46)
	Bilateral scapular arm elevation	0.82 (0.38/1.06)	0.86 (0.41/1.13)	0.65 (0.24/0.67)	0.79 (0.21/0.57)	0.63 (0.26/0.72)	0.88 (0.17/0.47)	0.86 (0.25/0.68)
	Bilateral frontal arm elevation	0.83 (0.45/1.23)	0.86 (0.39/1.07)	0.87 (0.12/0.33)	0.76 (0.29/0.80)	0.89 (0.11/0.31)	0.78 (0.26/0.71)	0.87 (0.23/0.65)
	Unilateral Sagittal arm elevation	0.93 (0.15/0.42)	0.84 (0.28/0.78)	0.79 (0.12/0.33)	0.93 (0.08/0.21)	0.55 (0.26/0.71)	0.89 (0.10/0.27)	x
	Unilateral Scapular arm elevation	0.90 (0.30/0.83)	0.91 (0.25/0.69)	0.81 (0.14/0.39)	0.88 (0.18/0.49)	0.84 (0.16/0.44)	0.81 (0.20/0.57)	x
Activities of daily living	Hair combing	0.89 (0.61/1.70)	0.95 (0.38/1.06)	x	x	x	x	x
	Low back washing	0.92 (0.44/1.21)	0.92 (0.51/1.41)	x	x	x	x	x
ICC (SEM/MDC) for inter-session intra-observer reliability								
Analytic arm movement	Bilateral sagittal arm elevation	0.40 (0.51/1.41)	0.59 (0.36/0.99)	0.34 (0.24/0.67)	0.42 (0.29/0.79)	0.24 (0.22/0.62)	0.69 (0.17/0.46)	0.52 (0.25/0.70)
	Bilateral scapular arm elevation	0.56 (0.46/1.28)	0.75 (0.23/0.64)	0.67 (0.07/0.19)	0.39 (0.25/0.70)	0.72 (0.11/0.30)	0.66 (0.12/0.33)	0.76 (0.17/0.47)
	Bilateral frontal arm elevation	0.57 (0.36/0.99)	0.43 (0.32/0.88)	0.49 (0.10/0.29)	0.52 (0.25/0.69)	0.44 (0.01/0.28)	0.47 (0.16/0.44)	0.59 (0.19/0.52)
	Unilateral Sagittal arm elevation	0.34 (0.41/1.13)	0.57 (0.20/0.54)	0.55 (0.10/0.27)	0.19 (0.27/0.75)	0.38 (0.15/0.41)	0.66 (0.10/0.27)	x
	Unilateral Scapular arm elevation	0.60 (0.38/1.06)	0.76 (0.33/0.92)	0.55 (0.14/0.38)	0.57 (0.16/0.45)	0.47 (0.26/0.72)	0.70 (0.14/0.38)	x
		0.60 (0.38/1.66)	0.76 (0.33/2.05)	0.55 (0.14/0.75)	0.57 (0.16/0.64)	0.47 (0.26/0.77)	0.70 (0.14/0.72)	x
Activities of daily living	Hair combing	0.65 (0.88/2.44)	0.48 (0.72/2.01)	x	x	x	x	x
	Low back washing	0.44 (1.17/3.24)	0.49 (1.07/2.96)	x	x	x	x	x
ICC (SEM/MDC) for inter-session inter-observer reliability								
Analytic arm movement	Bilateral sagittal arm elevation	0.76 (0.27/0.76)	0.78 (0.35/0.97)	0.73 (0.09/0.25)	0.68 (0.17/0.46)	0.71 (0.11/0.31)	0.70 (0.22/0.61)	0.77 (0.21/0.59)
	Bilateral scapular arm elevation	0.36 (0.80/2.22)	0.45 (0.85/2.37)	0.62 (0.16/0.45)	0.22 (0.41/1.13)	0.28 (0.31/0.87)	0.47 (0.41/1.13)	0.51 (0.47/1.31)
	Bilateral frontal arm elevation	0.44 (1.06/2.95)	0.34 (1.38/3.83)	0.64 (0.21/0.58)	0.19 (0.63/1.76)	0.78 (0.15/0.41)	0.22 (0.74/2.06)	0.40 (0.81/2.24)
	Unilateral Sagittal arm elevation	0.70 (0.45/1.25)	0.61 (0.52/1.44)	0.73 (0.14/0.38)	0.63 (0.24/0.66)	0.51 (0.20/0.55)	0.67 (0.21/0.57)	x
	Unilateral Scapular arm elevation	0.68 (0.60/1.66)	0.58 (0.74/2.05)	0.41 (0.27/0.75)	0.73 (0.23/0.64)	0.46 (0.28/0.77)	0.67 (0.26/0.72)	x
Activities of daily living	Hair combing	0.82 (0.62/1.72)	0.89 (0.61/1.70)	x	x	x	x	x
	Low back washing	0.64 (1.33/3.69)	0.69 (1.32/3.66)	x	x	x	x	x
Intrasession repeatability was assed using ICC(3,1) and intersession reliability was assessed using ICC(2,1)								
SEM and MDC are expressed in *ms* ^−2^								
Minimum detectable changes were computed with 95*%* level of agreement								

**TABLE 3:** Mean values of jerk metrics for all arm movements, expressed in *ms* ^−2^.

Movements		Total jerk for the right arm	Total jerk for the left arm	Jerk for ascending phase of right arm elevation	Jerk for lowering phase of right arm elevation	Jerk for ascending phase of left arm elevation	Jerk for lowering phase of left arm elevation	Total jerk for the both arm
Analytic arm movement	Bilateral sagittal arm elevation	2.46	2.77	1.52	1.91	1.69	2.16	3.72
	Bilateral scapular arm elevation	2.65	2.98	1.58	2.09	1.76	2.37	4.01
	Bilateral frontal arm elevation	3.20	3.59	1.91	2.52	2.15	2.81	4.83
	Unilateral Sagittal right arm elevation	2.54	0.91	1.63	1.91	0.62	0.65	2.71
	Unilateral Sagittal left arm elevation	0.88	2.81	0.60	0.63	1.84	2.09	2.95
	Unilateral Scapular right arm elevation	2.72	0.90	1.60	2.16	0.60	0.66	2.88
	Unilateral Scapular left arm elevation	0.91	3.05	0.61	0.67	1.86	2.37	3.19
Activities of daily living	Hair combing, right arm	4.77	0.88	3.55	3.00	0.66	0.55	4.86
	Hair combing, left arm	0.90	5.19	3.72	0.96	0.66	0.64	5.27
	Low back washing, right arm	4.44	0.84	2.81	3.32	0.58	0.59	4.53
	Low back washing, left arm	0.84	5.11	0.56	0.60	3.27	3.82	5.19

### 3.3 Comparisons of Jerks

#### 3.3.1 Comparison of Right and Left Jerk

All jerk ratios (7 comparisons, Table 4), indicated that right jerk was less than left jerk. Right jerk was significantly less than left jerk during sagittal, scapular and frontal bilateral arm elevation (*p* = 9.91 × 10^−5^, *p* = 6.47 × 10^−4^ and *p* = 6.30 × 10^−3^, respectively) and sagittal and scapular unilateral arm elevation (*p* = 3.40 × 10^−3^ and *p* = 2.67 × 10^−3^, respectively). Right jerk was also significantly less than left jerk during functional arm movement (*p* = 1.36 × 10^−2^ for hair combing and *p* = 1.47 × 10^−2^ for low back washing).

**TABLE 4:** Comparisons of jerk metrics between the dominant (right) arm and the non-dominant (left) arm.

Arm movement		Average jerk ratio *J*_Left_/(*J*_Left_ + *J*_Right_)	Wilcoxon *p*-value	Acceptance level
Analytic movements	Sagittal Bilateral Elevation	0.530	9.91 × 10^−5^	<0.01
	Scapular Bilateral Elevation	0.535	6.47 × 10^−4^	<0.01
	Frontal Bilateral Elevation	0.529	6.30 × 10^−3^	<0.01
	Sagittal Unilateral Elevation	0.522	3.40 × 10^−3^	<0.01
	Scapular Unilateral Elevation	0.533	2.67 × 10^−3^	<0.01
Functional movements	Hair combing	0.535	1.36 × 10^−2^	<0.05
	Low back washing	0.551	1.47 × 10^−2^	<0.05

#### 3.3.2 Comparison of Concentric and Eccentric Phases of Arm Elevation

All jerk ratios (7 comparisons, Table 5) indicated that jerk was significantly reduced during the ascending phase (concentric mode) than lowering (eccentric) phase of arm elevation, during bilateral arm elevation in the sagittal, scapular and frontal planes (*p* = 2.81 × 10^−3^, *p* = 3.11 × 10^−4^ and *p* = 1.33 × 10^−4^, respectively) and during unilateral right and left arm elevation in the sagittal and scapular planes (*p* = 1.26 × 10^−2^, 2.12 × 10^−2^, 4.95 × 10^−5^ and 4.47 × 10^−5^, respectively).

**TABLE 5:** Comparisons of jerk metrics between ascending and lowering phases of bilateral and unilateral arm elevation.

Arm movement	Average jerk ratio *J*_Ascending_/(*J*_Lowering_ + *J*_Ascending_)	Wilcoxon *p*-value	Acceptance level
Bilateral sagittal arm elevation	0.447	2.81 × 10^−3^	<0.01
Bilateral scapular arm elevation	0.432	3.11 × 10^−4^	<0.01
Bilateral frontal arm elevation	0.426	1.33 × 10^−4^	<0.01
Unilateral sagittal right arm elevation	0.460	1.26 × 10^−2^	<0.05
Unilateral sagittal left arm elevation	0.467	2.12 × 10^−2^	<0.05
Unilateral scapular right arm elevation	0.428	4.95 × 10^−5^	<0.01
Unilateral scapular left arm elevation	0.436	4.47 × 10^−5^	<0.01

#### 3.3.3 Comparison of Planes of Arm Elevation

All jerk ratios (3 comparisons, Table 6) indicated that jerk was reduced during sagittal than scapular arm elevation and was reduced in the scapular than frontal plane. Jerk during bilateral arm elevation was significantly reduced in the sagittal and scapular planes than frontal plane (*p* = 4.67 × 10^−12^ and *p* = 6.48 × 10^−9^, respectively) and the sagittal than scapular plane (*p* = 2.60 × 10^−2^). Jerk during unilateral left arm elevation was significantly reduced in the sagittal than scapular plane (*p* = 3.73 × 10^−2^). Jerk metrics did not differ between sagittal and scapular unilateral right arm elevation.

**TABLE 6:** Comparisons of jerk metrics between the different planes during bilateral and unilateral arm elevation.

Arm movement	Elevation plane (1)/Elevation plane (2)	Average jerk ratio *J*_(1)_/(*J*_(1)_ + *J*_(2)_)	Wilcoxon *p*-value	Acceptance level
Bilateral arm elevation	Frontal plane/Sagittal plane	0.567	4.67 × 10^−12^	<0.01
	Frontal plane/Scapular plane	0.548	6.48 × 10^−9^	<0.01
	Sagittal plane/Scapular plane	0.480	2.60 × 10^−2^	<0.05
Unilateral arm elevation	Sagittal plane/Scapular plane right arm	0.489	>0.05
	Sagittal plane/Scapular plane left arm	0.478	3.73 × 10^−2^	<0.05

## 4 Discussion

The goal of this study was to assess and compare jerk metrics between different conditions of active analytic and functional arm movements in asymptomatic participants. Our results show that jerk was reduced on the dominant side, in the sagittal plane and during the ascending phase of arm elevation.

### 4.1 Reliability of the Jerk Metrics

The intra-session trial-to-trial repeatability of the jerk metrics was good to excellent for analytic arm movements (except for jerk during the ascending phase of left arm elevation in the sagittal plane) and excellent for functional movements (simulation of ADL: hair combing and low back washing), which shows that the smoothness of successive movements was repeatable. The inter-session reliability was poor to excellent for analytic arm elevation and fair to excellent for functional movements. SEM and MDC remained small nevertheless, they can be considered high regarding the mean jerk values. The repeatability and reliability of functional movements can be linked to the nature of the movements. ADL are by definition goal-directed and everyday movements. Movement repetition improves movement direction and speed and favors control of the trajectory (Mawase et al., 2018). A study that assessed the reliability of jerk metrics for 11 everyday tasks in asymptomatic participants showed excellent inter-session intra-observer reliability for 81% of the tasks (Engdahl and Gates, 2019). Two other studies showed good (Takada et al., 2006) to excellent (Germanotta et al., 2015) inter-session intra-observer reliability of jerk metrics in asymptomatic subjects and in patients with Friedreich’s ataxia, respectively. Nevertheless, the RoM assessed was restricted: chewing jaw movements (Takada et al., 2006), reaching and manipulation tasks (Engdahl and Gates, 2019), and planar reaching movement (Germanotta et al., 2015). Overall, in the current study, intra-observer reliability was not superior to inter-observer reliability. The differences in jerk metrics found between 2 sessions may be explained more by the inherent intra-subject variability than observer-dependent factors. Some of the inter-session variability in jerk metrics could be explained by variations in physiologic tremor. This action tremor present in every healthy subject has a frequency of 8–12 Hz in young adults and varies with physiologic states such as muscular fatigue or emotional state (McAuley and Marsden, 2000). We could not exclude that the physiologic state of the participants varied between the 2 sessions.

The results of the current study favor the use of jerk metrics for clinical examination and research of arm movements provided that movements are usual and factors of intra-subject variability are controlled.

### 4.2 Effect of Laterality on Jerk

The current study showed that arm movements performed with the right dominant arm were smoother than with the left arm during all arm movements: bilateral, unilateral analytic, and functional. This result agrees with our first hypothesis.

Handedness defined as side preference and efficiency depends on practice and experience, which improve motor learning (Serrien et al., 2006; Nielsen and Cohen, 2008; Mawase et al., 2018). Long-term preferential use may result in changes in muscle fiber composition and activity (Diederichsen et al., 2007). Preferential use improves both quantitative (muscle strength and endurance) and qualitative (coordination and level of coactivation of muscles, coordination of muscles and joints etc.) muscle parameters (Demura et al., 2010). Nevertheless, several studies of differences in muscle activity between the arms had controversial results (Adam et al., 1998; Diederichsen et al., 2007). Greater muscular strength resulting from preferential use of the dominant arm (Crosby and Wehbé, 1994; Incel et al., 2002) could ease the motor control and the smoothness of the movement.

Jerk metrics well describe differences between dominant and non-dominant sides. In case of unilateral shoulder pathology (the most common case), comparing both arms using jerk metrics can help to better define the objective of treatment.

### 4.3 Effect of Contraction Modes on Jerk

Our results showed increased jerk metrics for arm lowering versus arm ascending during bilateral and unilateral arm elevation in all planes of movement, which indicates that the eccentric phase of the movement is less smooth than the concentric phase. This result is consistent with our second hypothesis.

Eccentric contractions are associated with a slowing down effect, supported by the recruitment of viscoelastic structures and the residual lasting force from the stretching of the active muscle or fibers (Hessel et al., 2017). Such contractions involve decreased recruitment of motor units and discharge rate, whereas the slowing down requires fine motor control (Enoka, 1996; Fang et al., 2001; Ebaugh and Spinelli, 2010; Duchateau and Baudry, 2014). The lower electromyography activity during eccentric contractions may induce less control of the variation of velocity that could explain the increased jerk metrics (Hoppeler, 2016; Hody et al., 2019).

Jerk metrics differ according to the mode of muscle contraction. Most of shoulder musculoskeletal pathologies (i.e., gleno-humeral osteoarthritis, rotator cuff disease) involve shoulder muscles weakness that makes the lowering phase easier to perform than the ascending one (Hody et al., 2019). Measuring the jerk metrics for both phases can help to monitor progress in patients.

### 4.4 Effect of Plane of Elevation on Jerk

According to our hypothesis, elevation was smoother in the sagittal and scapular planes than the frontal plane (for both unilateral and bilateral elevation). Several studies found higher humero-thoracic and scapulo-thoracic muscle activity during arm elevation in the frontal plane than the scapular and sagittal planes, which showed the lowest muscle activity (Castillo-Lozano et al., 2014; Ishigaki et al., 2015). In the frontal plane, owing to the convexity of the thorax, the rotation and linear displacement of the scapula cannot ensure optimal bony and muscular alignment (Johnston, 1937; Ludewig et al., 2009).

In opposition to our hypothesis, arm elevation was smoother in the sagittal than scapular plane during bilateral and left unilateral arm elevation. In the scapular plane, muscle rotational forces are balanced to keep the axis of the humerus and scapula aligned (Johnston, 1937; Kapandji, 1980; Ludewig et al., 2009). The increase in saccades for arm movements in the scapular versus sagittal plane may be related to fine muscle performance to keep the humeral head centered and remaining in the same plane during arm elevation (Suprak et al., 2006; Diederichsen et al., 2007). Visual feedback may play a role in the smoothness of movement. Movements in the sagittal plane are most visible to participants when they are asked to look straight ahead, and visual feedback during arm movements is known to be an important source of information for guiding movements (Sarlegna et al., 2004). Jerk metrics were not significantly lower in the sagittal than scapular plane during right arm elevation. This result may be related to both hand dominance and habits (the scapular plane is used for many unilateral daily activities, including hair combing) (Sheikhzadeh et al., 2008).

Jerk metrics differ according to the plane of arm elevation. We found that the sagittal plane was the most favorable to motor control, so it should be preferred to the scapular plane to begin rehabilitation of arm elevation. Nevertheless, the preferential plane of arm movement could vary according to the musculoskeletal or neuro-muscular disorder (Ludewig et al., 2009; Roren et al., 2012, 2013).

### 4.5 Strength and Limitations of the Study

To our knowledge, this is the first study to quantify and compare jerk metrics during different conditions of a selection of arm movements (maximal active arm elevation and functional movements) based on conventional clinical examination of the shoulder. These movements explore the greatest shoulder RoM; nevertheless, the assessment of jerk metrics for analytic glenohumeral axial rotations is lacking and we have no information about the other joints of the upper limb that contribute to the arm movements. The data pipeline used in this paper (including segmentation) is fully automated, mathematically based and empirically stable but remains to be validated with a larger database. We assessed the intra-session repeatability and inter-session reliability of jerk metrics. Our measures remain to be validated by comparing them to standard optoelectronic measurements. The limited sample size, the homogeneity of the population (right-handed, health professionals or students, asymptomatic and relatively young) and the lack of randomization of the successive movements limit the representativeness of our results. The movements were not performed freely, their duration was imposed and the functional movements were constrained for the sake of standardization. This pilot study brings new information that need to be completed by larger studies including participants with musculoskeletal disorders.

## 5 Conclusion

In accordance with 2 of our hypotheses, jerk metrics were reduced during all arm movements with the dominant right arm, and during analytic arm movements for the concentric (ascending) phase and in the sagittal plane. The jerk ratio is a normalized indicator easy to interpret for clinicians who are used to comparing one arm to the other for a diagnosis. The protocol of measurement is clinician- and patient-friendly (20-min examination, cost-efficient, with an easy-to-use and fully wearable device without area restriction or occlusion problems). The development of a jerk database including data for asymptomatic subjects and patients with musculoskeletal or neuromuscular pathologies could be useful to specify deficiencies, better define the objectives of treatment and monitor progress in patients with various shoulder pathologies. Future studies should also assess jerk metrics in various situations and environments of work and sports. Using IMUs, jerk metrics could be a promising way to assess the quality of movement and define the characteristics of movement performance.

## Data Availability

The original contributions presented in the study are included in the article/Supplementary Material, further inquiries can be directed to the corresponding authors.
