# Supplementary material for: Assessing Smoothness of Arm Movements With Jerk: A Comparison of Laterality, Contraction Mode and Plane of Elevation. A Pilot Study
Source: Front Bioeng Biotechnol. 2022 Jan 21;9:782740. doi: 10.3389/fbioe.2021.782740 (PMC8814310; doi:10.3389/fbioe.2021.782740)
Supplement: Supplementary file 1 [file DataSheet2.pdf]

1 APPENDIX

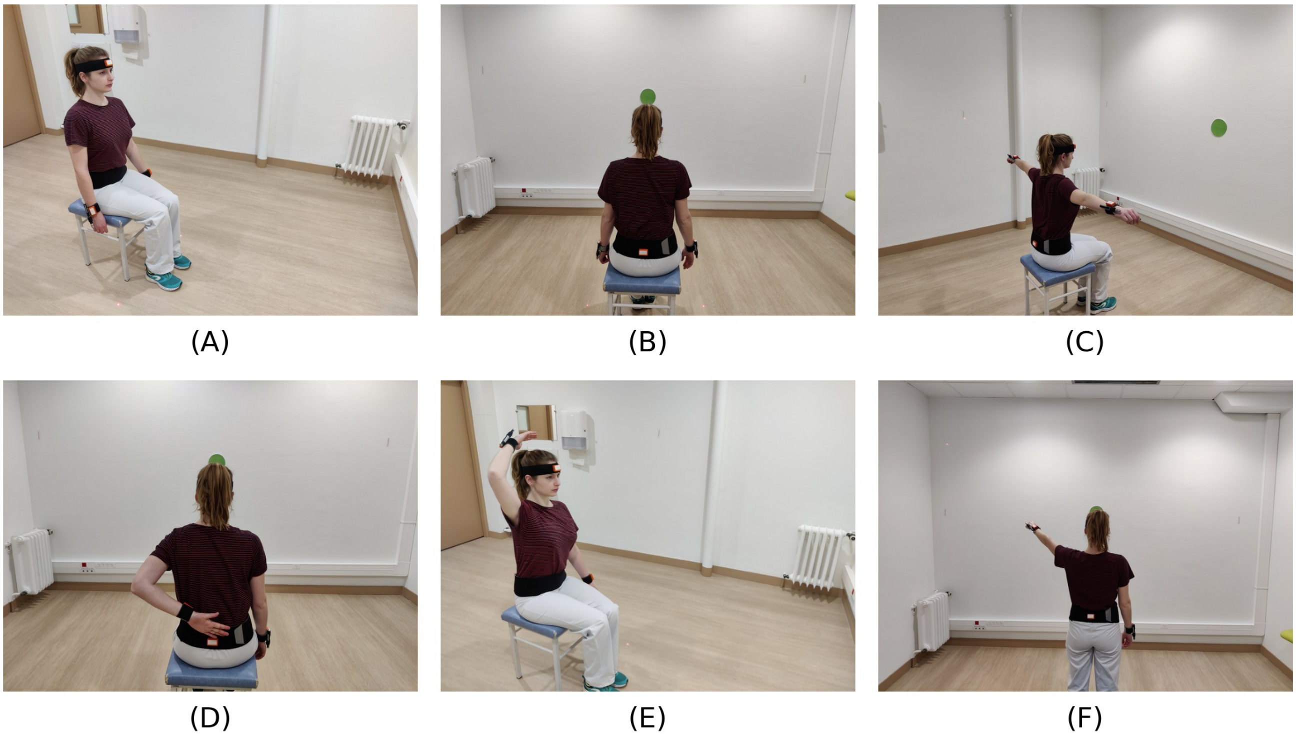

Figure A1. Illustration of a recording session. Rest position **(A)** (side view) and **(B)** (back view). Frontal bilateral arm elevation **(C)**, low-back washing **(D)**, hair combing **(E)** and unilateral scapular arm elevation **(F)**.

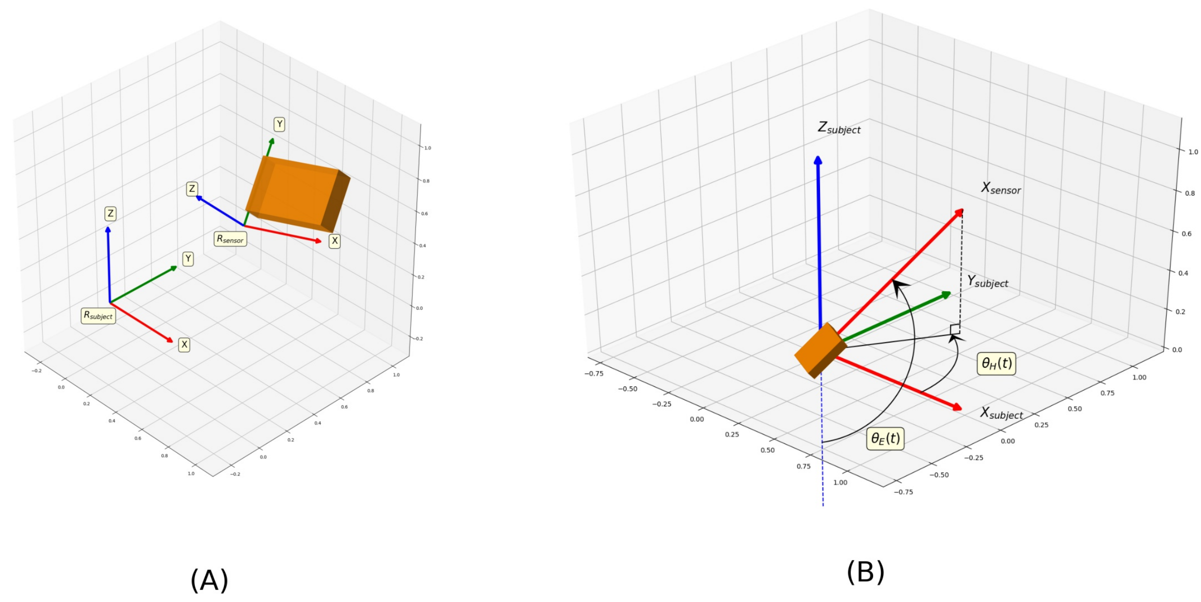

Figure A2. Sensor frame relative to the subject frame **(A)** and angles θ_z and θ_{xOy} of a sensor **(B)**. Colors red, green and blue correspond to axes X, Y and Z of the sensors, respectively.

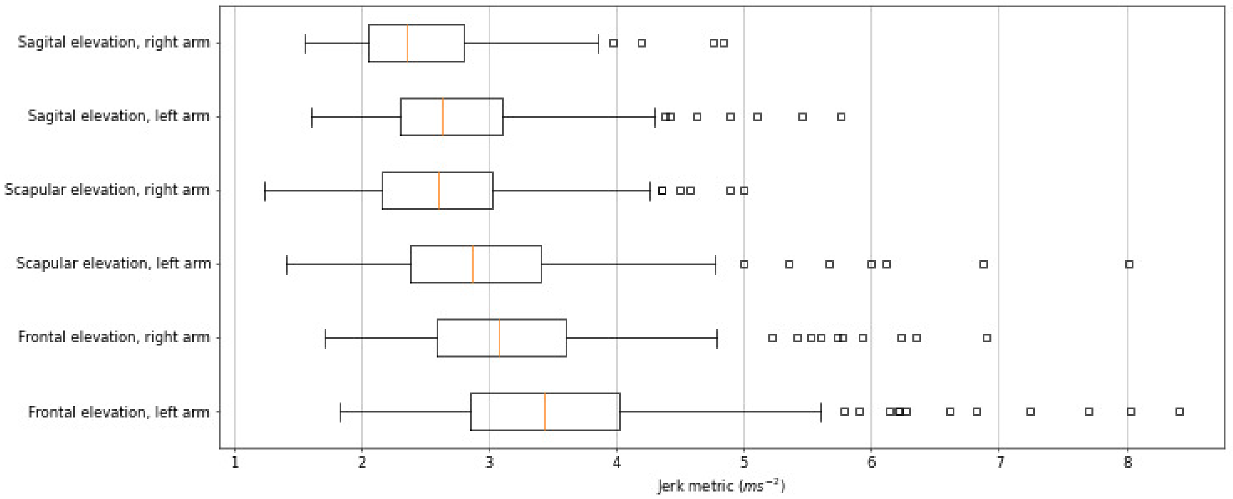

Figure A3. Box-plot of the jerk quantity for the right and left arm during arm elevations in the different planes. Data are median (vertical line), interquartile range (box edges) and range (whiskers).

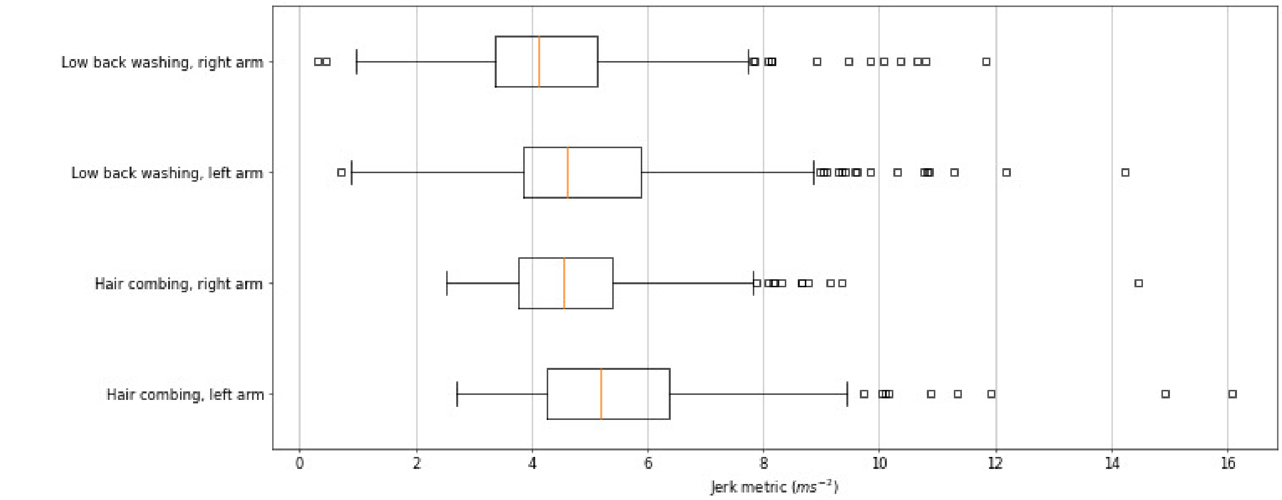

Figure A4. Box-plot of the jerk quantity for the right and left arm during arm elevations for functional movements. Data are median (vertical line), interquartile range (box edges) and range (whiskers).

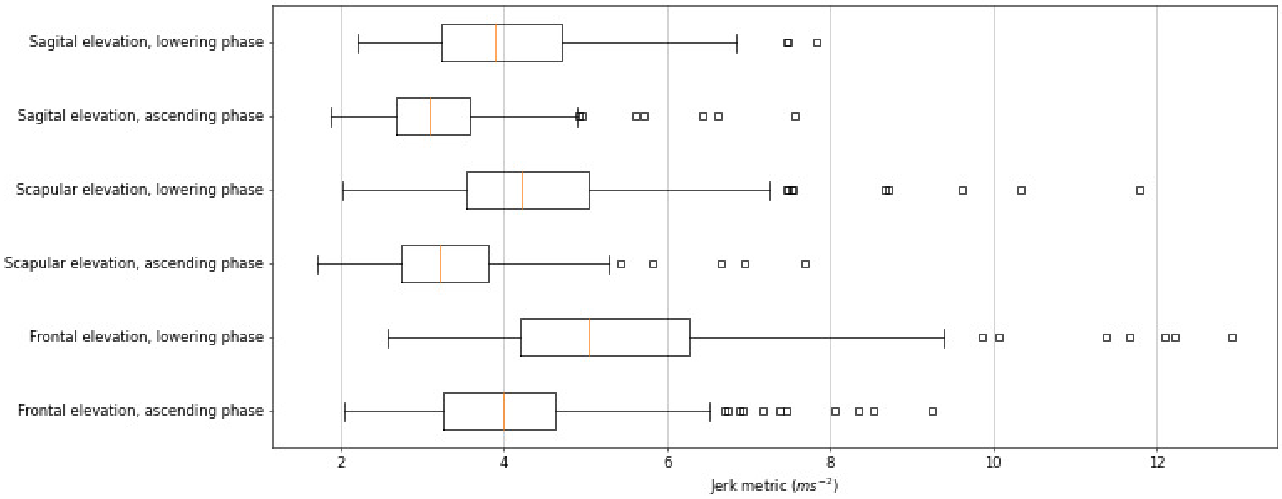

Figure A5. Box-plot of the jerk quantity for the ascending and lowering phase during arm elevations in the different planes. Data are median (vertical line), interquartile range (box edges) and range (whiskers).
